# Specialist Practices for Managing Persons Living with Dementia and Urinary Incontinence

**DOI:** 10.1007/s00192-025-06139-5

**Published:** 2025-04-29

**Authors:** Shweta A. Chawla, Krista L. Harrison, Louise C. Walter, Veronica Yank, Lufan Wang, Anne M. Suskind

**Affiliations:** 1 School of Medicine, University of California, San Francisco, 533 Parnassus Ave, San Francisco, CA USA; 2 Division of Geriatrics, Department of Medicine, University of California, San Francisco, San Francisco, CA USA; 3 Division of General Internal Medicine, Department of Medicine, University of California, San Francisco, San Francisco, CA USA; 4 Department of Urology, University of California, San Francisco, San Francisco, CA USA; 5 Division of Geriatrics, Department of Medicine, San Francisco Veterans Affairs Health Care System, San Francisco, CA USA; 6 Philip R. Lee Institute for Health Policy Studies at UCSF, San Francisco, CA USA

**Keywords:** Dementia, Patient management, Physician practices, Urinary incontinence

## Abstract

**Introduction and Hypothesis:**

Although persons living with dementia (PLWD) often experience urinary incontinence (UI), little is known about physician practice patterns across specialties treating these individuals. This study is aimed at assessing practice patterns across urologists, gynecologists, and geriatricians to highlight opportunities to support PLWD with UI and their caregivers.

**Methods:**

A vignette-based questionnaire was developed and sent to the members of the Society of Urodynamics, Female Pelvic Medicine & Urogenital Reconstruction (SUFU), the American Urogynecologic Society (AUGS), and the American Geriatrics Society (AGS) in this cross-sectional study. ANOVA and Chi-squared tests were used to compare responses between urologists, gynecologists, and geriatricians. Thematic analysis generated inductive themes from free-text responses.

**Results:**

The 273 respondents included 55 urologists, 173 gynecologists, and 45 geriatricians in the SUFU, AUGS, and AGS. Gynecologists and urologists were more likely to endorse treating PLWD with UI with beta-3 adrenergic receptor agonists and percutaneous tibial nerve stimulation and less likely to endorse discontinuing cholinesterase inhibitors than geriatricians (all *p* < 0.05). Geriatricians were more likely to agree with alleviating caregiver burden than urologists or gynecologists (all *p* < 0.05). Overall, 37% of urologists, 29% of gynecologists, and 61% of geriatricians felt prepared to care for these patients (*p* < 0.05). Thematic analysis of responses revealed four themes: medication changes, further work-up of symptoms, nonmedical care, and procedural interventions.

**Conclusions:**

This study identified differences in practice patterns between urologists, gynecologists, and geriatricians treating PLWD and UI, highlighting opportunities for cross-disciplinary learning to better care for these patients and their caregivers.

**Supplementary Information:**

The online version contains supplementary material available at 10.1007/s00192-025-06139-5

## Introduction

Dementia is a challenging medical condition that impairs independence in activities of daily living (ADLs) and affects both the person/s living with dementia (PLWD) and their family caregiver/s [1, 2]. Up to 90% of PLWD also experience urinary incontinence (UI), which increases caregiver burden [3, 4]. Studies have shown that family caregivers need greater guidance from healthcare professionals on managing incontinence and rate toileting needs as a primary contributor to caregiver burnout [5, 6].

Despite the frequent co-occurrence of dementia and UI, national and societal clinical guidelines for managing UI often do not account for comorbidities such as dementia. Many PLWD with UI are managed through care plans that address each condition separately and do not account for the complexity of implementing the plans, which usually require caregivers’ efforts [7]. As different physician specialties are attuned to different complexities, there is likely variation in care for these clinical issues [8]. Little is known, however, about how practices and beliefs vary across specialties of physicians who commonly care for patients with dementia and UI.

To address this gap, we designed a cross-sectional survey study to explore the practices and beliefs of urologists, gynecologists, and geriatricians in treating PLWD and UI [9, 10]. A vignette-based questionnaire was developed and distributed to members of three specialty societies: the Society of Urodynamics, Female Pelvic Medicine & Urogenital Reconstruction (SUFU), the American Urogynecologic Society (AUGS), and the American Geriatrics Society (AGS). This study aims to assess patient management practices across urologists, gynecologists, and geriatricians to highlight opportunities for cross-disciplinary learning and better understand how to support PLWD with UI and their caregivers.

## Materials and Methods

### Study Design

An online questionnaire was developed to assess physicians’ perspectives on the management of PLWD and UI. This questionnaire was distributed to SUFU, AUGS, and AGS members through society protocols (described in “Questionnaire Distribution”) and using the REDCap (Research Electronic Data Capture, Vanderbilt University, Nashville, TN, USA) platform [11, 12].

Approval to conduct the study was obtained from the Institutional Review Board (IRB# 22–37450), and the study qualified as exempt research [13].

### Questionnaire Design

The questionnaire, created in collaboration with clinical and research experts specializing in geriatric care, was pilot-tested with five urologists, urogynecologists, and geriatricians at the authors’ academic institution.

The questionnaire began with two screening questions to assess participants’ eligibility as board-certified physicians who treated patients aged 50 years and older in their practice. Demographic questions included the respondents’ identified specialty and subspecialty (whether they were trained in female pelvic medicine and reconstructive surgery [FPMRS], now called urogynecology and pelvic reconstructive surgery [URPS]), number of years of clinical experience, percentage of time spent providing clinical care, self-identified sex and gender, and region of the USA in which they practice, defined by national census divisions.

The questionnaire then presented a clinical vignette (Supplemental Table 1) with Likert-scale responses to assess respondents’ agreement with treatment options and perceived responsibility for the patient’s caregiver that reflected how they would manage the vignette patient and address caregiver burden in their clinical practices. An example of a treatment option included: “I would recommend percutaneous tibial nerve stimulation [for this patient],” which was followed by Likert-scale responses of “strongly agree,” “agree,” “neither agree nor disagree,” “disagree,” and “strongly disagree.” Closed-ended questions were followed by open-ended questions about additional management suggestions and factors considered when making treatment recommendations, which generated free-text data. Closed-ended questions and the number of respondents for each associated Likert-scale response are summarized in Tables 1 and 2. Additional closed-ended questionnaire items were aimed at understanding which resources respondents believed would help to care for PLWD with UI and whether respondents felt adequately prepared to care for these patients.

**Table 1:** Treatment recommendations by physicians in response to vignette—female person living with dementia with urinary incontinence whose husband has caregiver burden

Variable name	Total	Geriatricians (AGS)	Urologists (SUFU)	Gynecologists (AUGS)	*p* value
All subjects, *n* (%)	273 (100.0)	45 (16.5)	55 (20.1)	173 (63.4)	
I would recommend behavioral management (i.e., fluid management, avoiding bladder irritants, timed toileting)					0.7133
Strongly disagree	3 (1.4)	1 (2.5)	0 (0.0)	2 (1.5)	
Disagree	3 (1.4)	0 (0.0)	1 (2.3)	2 (1.5)	
Neither agree nor disagree (neutral)	9 (4.1)	0 (0.0)	3 (6.8)	6 (4.4)	
Agree	46 (20.9)	11 (27.5)	9 (20.5)	26 (19.1)	
Strongly agree	159 (72.2)	28 (70.0)	31 (70.5)	100 (73.5)	
Missing	53	5	11	37	
I would recommend a trial of a beta-3 adrenergic receptor agonist (e.g., mirabegron/Myrbetriq, vibegron/Gemtesa).					< 0.0001
Strongly disagree	5 (2.3)	1 (2.5)	0 (0.0)	4 (2.9)	
Disagree	10 (4.5)	9 (22.5)	0 (0.0)	1 (0.7)	
Neither agree nor disagree (neutral)	17 (7.7)	8 (20.0)	2 (4.6)	7 (5.1)	
Agree	78 (35.5)	17 (42.5)	15 (34.1)	46 (33.8)	
Strongly agree	110 (50.0)	5 (12.5)	27 (61.4)	78 (57.3)	
Missing	53	5	11	37	
I would recommend a trial of an antimuscarinic agent (such as trospium/Sanctura) that has a lower likelihood of cognitive side effects, compared with oxybutynin/Ditropan					0.2586
Strongly disagree	63 (28.6)	10 (25.0)	13 (29.6)	40 (29.4)	
Disagree	63 (28.6)	18 (45.0)	8 (18.2)	37 (27.2)	
Neither agree nor disagree (neutral)	35 (15.9)	6 (15.0)	8 (18.2)	21 (15.4)	
Agree	46 (20.9)	4 (10.0)	13 (29.6)	29 (21.3)	
Strongly agree	13 (5.9)	2 (5.0)	2 (4.6)	9 (6.6)	
Missing	53	5	11	37	
I would recommend 12 sessions of weekly percutaneous tibial nerve stimulation					0.0101
Strongly disagree	48 (21.8)	16 (40.0)	8 (18.2)	24 (17.7)	
Disagree	65 (29.6)	15 (37.5)	16 (36.4)	34 (25.0)	
Neither agree nor disagree (neutral)	51 (23.2)	6 (15.0)	9 (20.5)	36 (26.5)	
Agree	37 (16.8)	2 (5.0)	9 (20.5)	27 (19.1)	
Strongly agree	19 (8.6)	1 (2.5)	2 (4.6)	16 (11.8)	
Missing	53	5	11	37	
I would recommend talking with the physician prescribing the donepezil/Aricept					< 0.0001
Strongly disagree	40 (18.2)	2 (5.0)	5 (11.4)	33 (24.3)	
Disagree	47 (21.4)	3 (7.5)	13 (29.6)	31 (22.8)	
Neither agree nor disagree (neutral)	42 (19.1)	2 (5.0)	9 (20.5)	31 (22.8)	
Agree	59 (26.8)	14 (35.0)	15 (34.1)	30 (22.1)	
Strongly agree	32 (14.6)	19 (47.5)	2 (4.6)	11 (8.1)	
Missing	53	5	11	37	
I would discuss different types of continence care products with the patient and her husband					0.2029
Strongly disagree	6 (2.7)	3 (7.5)	0 (0.0)	3 (2.2)	
Disagree	14 (6.4)	2 (5.0)	1 (2.3)	11 (8.1)	
Neither agree nor disagree (neutral)	36 (16.4)	7 (17.5)	6 (13.6)	23 (16.9)	
Agree	95 (43.2)	20 (50.0)	23 (52.3)	52 (38.2)	
Strongly agree	69 (31.4)	8 (20.0)	14 (31.8)	47 (34.6)	
Missing	53	5	11	37	
I would tell the husband that I do not think that any treatment is warranted					0.0674
Strongly disagree	85 (38.6)	23 (57.5)	11 (25.0)	51 (37.5)	
Disagree	97 (44.1)	13 (32.5)	21 (47.7)	63 (46.3)	
Neither agree nor disagree (neutral)	24 (10.9)	2 (5.0)	6 (13.6)	16 (11.8)	
Agree	11 (5.0)	1 (2.5)	5 (11.4)	5 (3.7)	
Strongly agree	3 (1.4)	1 (2.5)	1 (2.3)	1 (0.7)	
Missing	53	5	11	37	

*AGS* American Geriatrics Society, *SUFU* Society of Urodynamics, Female Pelvic Medicine & Urogenital Reconstruction, *AUGS* American Urogynecologic Society

**Table 2:** Further management recommendations by physicians and factors influencing decision-making in response to vignette—female person living with dementia with urinary incontinence whose husband has caregiver burden

Variable name	Total	Geriatricians (AGS)	Urologists (SUFU)	Gynecologists (AUGS)	*p* value
All Subjects, *n* (%)	273 (100.0)	45 (16.5)	55 (20.1)	173 (63.4)	
I would make a referral to a social worker					0.4059
Strongly disagree	13 (5.9)	0 (0.0)	4 (9.1)	9 (6.6)	
Disagree	27 (12.3)	3 (7.5)	7 (15.9)	17 (12.5)	
Neither agree nor disagree (neutral)	42 (19.1)	5 (12.5)	8 (18.2)	29 (21.3)	
Agree	87 (39.6)	21 (52.5)	16 (36.4)	50 (36.8)	
Strongly agree	51 (23.2)	11 (27.5)	9 (20.5)	31 (22.8)	
Missing	53	5	11	37	
I would connect the husband to support groups					0.0055
Strongly disagree	9 (4.1)	1 (2.5)	2 (4.6)	6 (4.4)	
Disagree	27 (12.3)	1 (2.5)	9 (20.5)	17 (12.5)	
Neither agree nor disagree (neutral)	54 (24.6)	4 (10.0)	13 (29.6)	37 (27.2)	
Agree	75 (34.1)	16 (40.0)	16 (36.4)	43 (31.6)	
Strongly agree	55 (25.0)	18 (45.0)	4 (9.1)	33 (24.3)	
Missing	53	5	11	37	
I would connect the patient to support groups					0.0886
Strongly disagree	37 (16.8)	11 (27.5)	6 (13.6)	20 (14.7)	
Disagree	66 (30.0)	9 (22.5)	16 (36.4)	41 (30.2)	
Neither agree nor disagree (neutral)	61 (27.7)	6 (15.0)	16 (36.4)	39 (28.7)	
Agree	39 (17.7)	11 (27.5)	5 (11.4)	23 (16.9)	
Strongly agree	17 (7.7)	3 (7.5)	1 (2.3)	13 (9.6)	
Missing	53	5	11	37	
I would ask other members of the health care team to follow					0.2954
Strongly disagree	23 (10.4)	5 (12.5)	4 (9.1)	14 (10.3)	
Disagree	46 (20.9)	5 (12.5)	11 (25.0)	30 (22.1)	
Neither agree nor disagree (neutral)	42 (19.1)	5 (12.5)	12 (27.3)	25 (18.4)	
Agree	74 (33.6)	14 (35.0)	13 (29.6)	47 (34.6)	
Strongly agree	35 (15.9)	11 (27.5)	4 (9.1)	20 (14.7)	
Missing	53	5	11	37	
It is my responsibility as a doctor to treat the patient, not the caregiver [husband]					<.0001
Strongly disagree	38 (18.3)	16 (41.0)	13 (31.0)	9 (7.1)	
Disagree	81 (38.9)	17 (43.6)	17 (40.5)	47 (37.0)	
Neither agree nor disagree (neutral)	38 (18.3)	2 (5.1)	4 (9.5)	32 (25.2)	
Agree	37 (17.8)	2 (5.1)	7 (16.7)	28 (22.1)	
Strongly agree	14 (6.7)	2 (5.1)	1 (2.4)	11 (8.7)	
Missing	65	6	13	46	
It is my responsibility as a doctor to treat the patient and to alleviate the caregiver’s burden					0.0007
Strongly disagree	2 (1.0)	0 (0.0)	1 (2.4)	1 (0.8)	
Disagree	2 (1.0)	1 (2.6)	0 (0.0)	1 (0.8)	
Neither agree nor disagree (neutral)	13 (6.3)	0 (0.0)	2 (4.8)	11 (8.7)	
Agree	106 (51.2)	10 (25.6)	20 (47.6)	76 (60.3)	
Strongly agree	84 (40.6)	28 (71.8)	19 (45.2)	37 (29.3)	
Missing	66	6	13	47	
The fact that the caregiver [husband] is overwhelmed factored into my treatment decision/s					0.0044
Strongly disagree	1 (0.5)	0 (0.0)	1 (2.4)	0 (0.0)	
Disagree	1 (0.5)	0 (0.0)	0 (0.0)	1 (0.8)	
Neither agree nor disagree (neutral)	10 (4.8)	0 (0.0)	3 (7.1)	7 (5.6)	
Agree	115 (55.6)	13 (33.3)	22 (52.4)	80 (63.5)	
Strongly agree	80 (38.7)	26 (66.7)	16 (38.1)	38 (30.2)	
Missing	66	6	13	47	
The fact that the husband considered placing the patient in a nursing facility because of her incontinence factored into my treatment decision/s					0.1213
Strongly disagree	3 (1.4)	0 (0.0)	1 (2.4)	2 (1.6)	
Disagree	12 (5.8)	1 (2.6)	0 (0.0)	11 (8.7)	
Neither agree nor disagree (neutral)	22 (10.6)	1 (2.6)	6 (14.3)	15 (11.8)	
Agree	89 (42.8)	15 (38.5)	19 (45.2)	55 (43.3)	
Strongly agree	82 (39.4)	22 (56.4)	16 (38.1)	44 (34.7)	
Missing	65	6	13	46	
I felt adequately prepared by training to care for patients with dementia/cognitive impairment and bladder-related issues					0.2107
Yes	97 (48.0)	24 (63.2)	19 (47.5)	54 (43.6)	
Somewhat	87 (43.1)	13 (34.2)	16 (40.0)	58 (46.8)	
No	18 (8.9)	1 (2.6)	5 (12.5)	12 (9.7)	
Missing	71	7	15	49	
I felt adequately prepared by training to care for complex situations related to caregivers of patients with dementia/cognitive impairment and bladder-related issues					0.0035
Yes	73 (36.0)	23 (60.5)	15 (36.6)	35 (28.2)	
Somewhat	94 (46.3)	13 (34.2)	16 (39.0)	65 (52.4)	
No	36 (17.7)	2 (5.3)	10 (24.4)	24 (19.4)	
Missing	70	7	14	49	

*AGS* American Geriatrics Society, *SUFU* Society of Urodynamics, Female Pelvic Medicine & Urogenital Reconstruction, *AUGS* American Urogynecologic Society

### Questionnaire Distribution

The research team coordinated with representatives from each society to distribute questionnaires to their members using standard protocols.

The questionnaire was distributed via email to the SUFU membership on 4 April 2023 and the AGS membership on 28 April 2023. For AUGS, the questionnaire was distributed on 23 August 2023, and two reminders were sent to encourage participants to complete the questionnaire by 3 October 2023. According to reports from representatives of each specialty society, there were 726 individuals on the SUFU listserv, 6032 individuals on the AUGS listserv, and 8919 individuals on the AGS listserv. However, each of these listservs includes heterogeneous populations of clinicians, scientists, advanced practice providers, trainees, and other types of members; therefore, it is impossible to discern the true denominator of individuals who were eligible to complete the questionnaire. Demographic data on nonresponders were not collected, as each organization did not provide these data. Late questionnaires were accepted until 17 October 2023. No incentives were offered for completing the questionnaire.

### Data Analysis

Statistical analysis of multiple-choice responses was performed using SAS 9.4. Chi-squared or Fisher’s exact tests were used to determine differences between physician subgroups when analyzing categorical variables; *t*-tests were used with continuous variables. Missing data were removed using pairwise deletion.

Free-text questionnaire responses were exported from REDCap and organized using qualitative content analysis; similar language was noted for initial inductive codes, which were placed into broader categories of subthemes and themes. Initial analysis was conducted independently by three authors (SAC, KLH, AMS). Themes and subthemes were then compared between authors and reconciled through discussions that included authors SAC, AMS, VY, and LCW.

## Results

### Quantitative Responses

Among the 319 respondents, 32 were excluded for not identifying as physicians, given differences in average years and metrics for formal training, and 12 did not respond to any questions regarding patient management.

The final sample included 273 respondents. The most common specialty represented was gynecologists (63.4%), followed by urologists (20.1%) and geriatricians (16.5%). Most physicians who responded to demographic questions (62.5%) had been practicing in their identified specialty for at least 10 years, and 49.1% of these physicians identified as female/women.

Among urologists and gynecologists, 65.5% and 70.5%, respectively, were subspecialty board-certified in URPS. In the questionnaire’s initial question (Q1), when respondents were asked to identify their specialty, many respondents noted that they were initially trained in urology, gynecology, geriatrics, primary care, or internal medicine. This question also included the option of “Other” and the ability to write in free text. Respondents could further clarify if they were trained in FPMRS (now URPS) in a follow-up question. However, 63 respondents chose the “Other” option in Q1 and identified themselves as urogynecologists in the free-text response. Thus, their responses did not indicate their initial specialty training (urology or gynecology). Sixty-one respondents in this group completed the questionnaire when it was distributed to the AUGS listserv, after the response period for members on the SUFU and AGS listservs had ended; thus, these respondents were categorized as gynecologists.

Two respondents did not self-identify their residency training and identified as urogynecologists when the questionnaire was distributed to the AGS; these respondents were removed from the analysis, as they could not be reliably categorized as initially trained in urology or gynecology. The results of the surveyed demographic data are summarized in Table 3.

**Table 3:** Demographics and training of physician sample

Variable name	Total	Geriatricians	Urologists	Gynecologists
All subjects, *n* (%)	273 (100.0)	45 (16.5)	55 (20.1)	173 (63.4)
Years of practice				
I am currently a resident	1 (0.5)	0 (0.0)	0 (0.0)	1 (0.8)
I am currently a fellow	14 (6.9)	1 (2.6)	0 (0.0)	13 (10.5)
1–5	35 (17.2)	8 (21.1)	3 (7.3)	24 (19.4)
5–10	26 (12.8)	8 (21.1)	6 (14.6)	12 (9.7)
10–20	59 (29.1)	4 (10.5)	16 (39.0)	39 (31.5)
20+	68 (33.5)	17 (44.7)	16 (39.0)	35 (28.2)
Missing	70	7	14	49
Percentage of clinical time in practice				
0–25%	6 (3.0)	4 (10.5)	0 (0.0)	2 (1.6)
25–50%	17 (8.5)	5 (13.2)	0 (0.0)	12 (9.8)
50–75%	65 (32.3)	18 (47.4)	15 (36.6)	32 (26.2)
75–100%	113 (56.2)	11 (29.0)	26 (63.4)	76 (62.3)
Missing	72	7	14	51
Subspecialty board certified or eligible in urogynecology and pelvic reconstructive surgery among eligible participants				
Yes	158 (69.3)	–	36 (65.5)	122 (70.5)
No	13 (5.7)	–	5 (9.1)	8 (4.6)
In training	9 (3.9)	–	0 (0.0)	9 (5.2)
Missing	48	–	14	34
Gender identity				
Male/man	65 (23.8)	13 (28.9)	15 (27.3)	37 (21.4)
Female/woman	134 (49.1)	25 (55.6)	25 (45.5)	84 (48.6)
Other				
Decline to state	4 (1.5)	0 (0.0)	1 (1.8)	3 (1.7)
Missing	70	7	14	49
United States region				
West	55 (27.1)	11 (29.0)	13 (31.7)	31 (25.0)
Midwest	45 (22.2)	9 (23.7)	7 (17.1)	29 (23.4)
Northeast	53 (26.1)	8 (21.1)	10 (24.4)	35 (28.2)
South	50 (24.6)	10 (26.3)	11 (26.8)	29 (23.4)
Missing	70	7	14	49

Table 1 shows specialist treatment preferences in response to the vignette, in which a female PLWD taking a cholinesterase inhibitor for dementia is experiencing increased urinary incontinence, which has been overwhelming her primary caregiver. Although 95.5% of urologists and 91.1% of gynecologists agreed with recommending beta-3 adrenergic receptor agonists, only 55% of geriatricians agreed with prescribing these medications (*p* < 0.001). Among respondents, 25.1% of urologists and 30.9% of gynecologists agreed with recommending percutaneous tibial nerve stimulation, compared with 7.5% of geriatricians (*p* = 0.01). Compared with 38.7% of urologists and 30.2% of gynecologists, 82.5% of geriatricians agreed with considering alternative dementia management options for the patient, as donepezil can worsen UI (*p* < 0.001). Physicians’ Likert-scale responses to additional treatments and management strategies are summarized in Table 1.

Participant responses to questions regarding perceived responsibility are summarized in Table 2. Compared with urologists and gynecologists, geriatricians were more likely to agree with connecting the caregiver with support groups (*p* = 0.006), agree that they had a responsibility to alleviate caregiver burden (*p* = 0.001), and factor in caregivers’ feelings of being overwhelmed in treatment decisions (*p* = 0.004). Gynecologists were more likely to agree that their responsibility was to treat the patient, not the caregiver (*p* < 0.0001). Among respondents, 47.5% of urologists, 43.6% of gynecologists, and 63.2% of geriatricians agreed that their training adequately prepared them to care for PLWD with bladder-related issues. More geriatricians (60.5%) agreed that their training had adequately prepared them to care for complex situations related to caregivers of PLWD with UI than urologists (36.6%) and gynecologists (28.2%; *p* = 0.004).

A secondary analysis comparing urogynecologists with urologists and gynecologists without URPS training showed similarities in responses among the three groups, with statistically significant differences in responses to only two questions: first, “The fact that the caregiver [husband] is overwhelmed factored into my treatment decision/s” (*p* = 0.024), and second, “The fact that the husband considered placing the patient in a nursing facility because of her incontinence factored into my treatment decision/s” (*p* = 0.013; Supplemental Table 2).

When considering resources for caring for PLWD with incontinence, 22.8% of urologists and gynecologists supported reviewing relevant research, 16.7% supported participating in topical online training, 14.9% supported attending a topical online conference, and 40.4% supported attending an in-person conference. Five percent of specialists supported being presented with guidelines to inform their management of PLWD with incontinence.

### Qualitative Analysis

Of the 273 completed questionnaires, 82 respondents provided 111 free-text comments representing urologists (19), gynecologists (65), and geriatricians (27). Thematic saturation was 97.4% [14].

Four overall themes emerged from responses regarding the vignette:

1. Medication changes
2. Further work-up
3. Nonmedical care
4. Procedural or invasive interventions

**Fig. 1:**
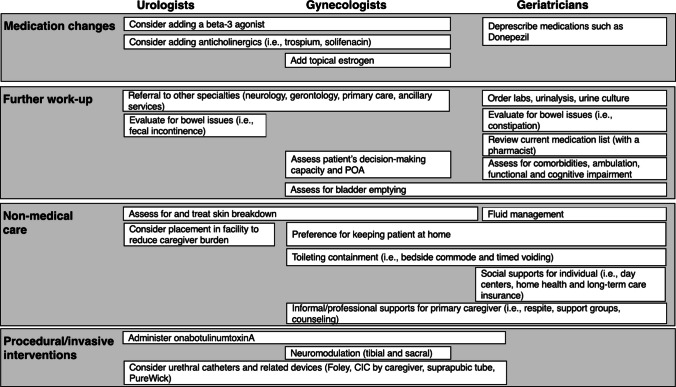
Themes and sub-themes of physician responses for proposed additional treatments. *POA* power of attorney, *CIC* clean intermittent catheterization

Themes and sub-themes are presented in Fig. 1.

#### Theme 1: Medication Changes

Physicians discussed medical management in 15 responses. Consistent with the results of the quantitative responses, urologists and gynecologists recommended medications such as beta-3 adrenergic receptor agonists and anticholinergics, whereas geriatricians suggested deprescribing donepezil. Gynecologists further suggested adding topical estrogen for symptom management.

#### Theme 2: Further Work-Up

Thirty responses recommended further diagnostic work-up. Urologists and gynecologists proposed referrals to physicians from other specialties for management. For example, one gynecologist noted limitations in staffing:

“My practice does not have social worker access. We do not manage dementia and would redirect to the PCP.” [Gynecologist 30].

A urologist said:

“These are issues for the PCP.” [Urologist 2].

Gynecologists also suggested tracking the patient’s continence with voiding diaries. Urologists uniquely proposed evaluating patients for fecal incontinence. Both gynecologists and geriatricians suggested assessing for urinary retention, and geriatricians recommended ordering urinalyses and urine cultures.

Gynecologists also emphasized the importance of assessing the patient’s decision-making capacity; assessments of the patient’s capacity were included in the theme of further work-up to determine the impact of the patient’s dementia on her medical autonomy. One gynecologist noted how the determination of capacity could affect treatment considerations:

“Some of this [work-up] depends on who makes her medical decisions. If she is still competent to do so, I would be less aggressive. If not, then I think [her caregiver] should have a little more input into these decisions as she may not be in her best mental state to say that she is not bothered.” [Gynecologist 35]

#### Theme 3: Nonmedical Care

In 33 responses, physicians recommended “nonmedical care,” which included alleviating caregiver burden or the patient’s symptoms without medical or procedural interventions. Urologists and gynecologists suggested treating skin breakdowns resulting from incontinence. Gynecologists and geriatricians recommended toileting containment strategies such as timed voiding and bedside commodes. Urologists, gynecologists, and geriatricians discussed how caregiver burden could impact the patient’s placement in a nursing home:

“I cannot push medications or treatments on a patient who cannot consent and whose diapers can be managed if not by the husband but by a carer inhouse or at a home. New normals can be hard but are inevitable.” [Gynecologist 43]

“This is not a fixable problem. [The caregiver’s] life will improve by [bringing] her to a care facility.” [Urologist 11]

“By treating incontinence that doesn't bother her but keeps her out of a home, you are still treating her because her mental health will benefit by not being separated from her family.” [Gynecologist 36]

Other responses considered support for the caregiver:

“Patient care is not isolated to the patient.” [Urologist 4]

“Husband needs support group and counseling.” [Geriatrician 12]

#### Theme 4: Procedural or Invasive Interventions

In 33 responses, urologists and gynecologists suggested procedural interventions such as onabotulinumtoxinA injections, clean intermittent catheterization placement, Foley catheters, and PureWick catheters. Gynecologists also suggested sacral and tibial nerve neuromodulation. Geriatricians did not propose procedural or invasive interventions.

## Discussion

This study examines the practice patterns of urologists, gynecologists, and geriatricians who treat PLWD with UI. Among the study’s respondents, urologists and gynecologists were more likely to recommend *adding* treatments (i.e., beta-3 adrenergic receptor agonists, onabotulinumtoxinA, neuromodulation, urinary catheters) to the patient’s regimen, whereas geriatricians were more likely to *deprescribe* treatments (i.e., donepezil). Geriatricians were also more likely to feel responsible for managing caregiver burden and to consider it in their treatment plans, consistent with prior research [15].

Thematic analysis of the free-text responses revealed additional factors that influence treatment decisions. Although previous literature has highlighted the roles of urologists and gynecologists in evaluating and treating fecal incontinence, only urologists suggested evaluating fecal incontinence [16, 17]. Although gynecologists considered assessing patient decision-making capacity, urologists and geriatricians did not discuss this in their responses, despite studies showing the significant role that geriatricians play when determining decision-making capacity [18, 19]. Previous studies also indicate that, when developing treatment plans for PLWD with UI, shared decision-making with physicians only included the patient’s caregiver [20–22]. Furthermore, although the questionnaire did not directly assess respondents’ opinions on institutionalization for the PLWD, free-text responses from gynecologists and geriatricians addressed how their treatment decisions were influenced by the aim of keeping the patient in her home. However, only one urologist discussed this topic in the free-text responses and suggested placing the patient in a care facility. Whereas the role of incontinence as a predictor of placement in a long-term care facility has been shown in some previous studies, especially if other comorbidities are present, these studies have not addressed how this is influenced by the treating physicians’ recommendations [23–25]. These results indicate areas for further research to determine which clinical and psychosocial factors physicians consider when recommending management plans for PLWD with UI; however, as the qualitative analysis focused on responses to open-ended questions, these results are only representative of the subset of respondents who were analyzed, further underscoring the need for future studies.

Findings from the qualitative analyses that reflected the findings of the quantitative analyses included the concordance between the responses of gynecologists and urologists and the differences between the recommendations of these specialties compared with those of geriatricians. Previous literature supports the role of gynecologists as both primary care providers and surgeons, whereas urologists have generally been considered to be surgical specialists; the concordance between the respondents of these specialties could be due to their training and academic interests, including a surgical career focus, of the subsets of each specialty in the AUGS and the SUFU [26, 27].

The management of PLWD with UI requires further educational resources. Although only 5% of the urologists and gynecologists surveyed supported being presented with guidelines to inform their management of these patients, research has shown that clinical guidelines can alleviate uncertainty in clinical dilemmas and may help to reconcile specialty-specific differences by integrating the clinical approaches of different specialists. Previous guidelines considering the use of anticholinergic medications and the associated risk of dementia considered procedural interventions and beta-3 agonists for managing overactive bladder symptoms [28]. However, future guidelines should incorporate the perspectives of specialists and primary care providers, consider multiple possible interventions for managing PLWD with UI, and apply to various clinical settings, including those with and without social workers and care navigators [29, 30]. Additionally, the evolving evidence of the efficacy of interventions for UI should be iteratively updated.

Limitations of this study include the dissemination of the questionnaire by email, whereby respondents using email blockers and members with incorrect email addresses may never have received the questionnaire. Furthermore, the heterogeneous populations of clinicians, scientists, advanced practice providers, trainees, and other types of members on each society’s listserv also contributed to the inability to reliably calculate a response rate among eligible respondents, which should be considered when interpreting these data. As some respondents identified themselves as urogynecologists but did not indicate their initial training in urology or gynecology, the timing of their questionnaire responses was used to categorize them as gynecologists and may have led to misclassification of these individuals. Furthermore, variation in the distribution approaches used by the societies could have resulted in differential reach for the targeted specialist groups, and representatives of the societies were unable to provide demographic information about their membership pool to compare those who responded to the questionnaire with nonresponders. Physician responses may also not reflect their real-world practice patterns, an inherent bias to many survey designs.

This study highlights significant differences in how urologists, gynecologists, and geriatricians manage PLWD with UI, underscoring the need for interdisciplinary collaboration, education, and research to develop comprehensive care plans for these patients and their caregivers.

## Supplementary Information

Below is the link to the electronic supplementary material.

Supplementary file1 (DOCX 29 KB)

## Data Availability

Data that support the findings of this study include participants' demographic information and are available from the corresponding author upon reasonable request.
