# Supplementary material for: Specialist Practices for Managing Persons Living with Dementia and Urinary Incontinence
Source: Int Urogynecol J. 2025 Apr 29;36(7):1473–83. doi: 10.1007/s00192-025-06139-5 (PMC12356740; doi:10.1007/s00192-025-06139-5)
Supplement: Supplementary file 1 — Supplementary file1 (DOCX 29 KB) [file 192_2025_6139_MOESM1_ESM.docx]

**Supplemental Table 1: Vignette Descriptions**

| **Vignette 1** |
| --- |
| A 73-year-old patient (she/her) presents to your office with moderate Alzheimer's dementia and urinary frequency, urgency, and associated incontinence. She is accompanied by her husband, who is her primary caregiver. She has been taking Donepezil (Aricept) for her dementia for 5 years, which has an associated risk of increasing her urinary incontinence. Her urinary incontinence is currently managed with a diaper. She notes that she is not bothered by her symptoms and does not feel that further interventions are needed to manage her incontinence. However, her husband is overwhelmed and has difficulty caring for her urinary issues at home and consistently changing her diapers. He is considering transferring her to a nursing facility if her symptoms cannot be improved but hopes to avoid this outcome. He asks for your guidance on possible next steps of her management. |

**Supplemental Table 2**

*Treatment and Management Recommendations by Urogynecologists Compared to Urologists and Gynecologists without URPS training in Response to Vignette –Female Person Living with Dementia with*

*Urinary Incontinence whose Husband has Caregiver Burden*

| **Variable Name** | **Total** | |  | **Urologists (no URPS training)** | **Gynecologists**  **(No URPS training)** | **Urogynecologists** | **P value** |
| --- | --- | --- | --- | --- | --- | --- | --- |
| **All Subjects (n) (%)** | 228 (100.0) | |  | 19 (8.3) | 42 (18.4) | 167 (73.3) |  |
| **I would recommend behavioral management (i.e., fluid management, avoiding bladder irritants, timed toileting).** | | | | | | | 0.1504 |
| Strongly disagree | 2 (1.1) | |  | 0 (0.0) | 1 (7.2) | 1 (0.6) |  |
| Disagree | 3 (1.7) | |  | 0 (0.0) | 0 (0.0) | 3 (1.9) |  |
| Neither agree nor disagree (neutral) | 9 (5.0) | |  | 0 (0.0) | 1 (7.2) | 8 (5.1) |  |
| Agree | 35 (19.4) | |  | 4 (50.0) | 4 (28.6) | 27 (17.1) |  |
| Strongly agree | 131 (72.8) | |  | 4 (50.0) | 8 (57.1) | 119 (75.3) |  |
| Missing | 48 | |  | 11 | 28 | 9 |  |
| **I would recommend a trial of a Beta-3 adrenergic receptor agonist (e.g. Mirabegron/Mirabetriq, Vibegron/Gemtesa).** | | | | | | | 0.8754 |
| Strongly disagree | 4 (2.2) | |  | 0 (0.0) | 0 (0.0) | 4 (2.5) |  |
| Disagree | 1 (0.6) | |  | 0 (0.0) | 0 (0.0) | 1 (0.6) |  |
| Neither agree nor disagree (neutral) | 9 (5.0) | |  | 0 (0.0) | 2 (14.3) | 7 (4.4) |  |
| Agree | 61 (33.9) | |  | 3 (37.5) | 4 (28.6) | 54 (34.2) |  |
| Strongly agree | 105 (58.3) | |  | 5 (62.5) | 8 (57.1) | 92 (58.2) |  |
| Missing | 48 | |  | 11 | 28 | 9 |  |
| **I would recommend a trial of an antimuscarinic agent (such as Trospium/Sanctura) that has a lower likelihood of cognitive side effects, compared to Oxybutynin/Ditropan.** | | | | | | | 0.3696 |
| Strongly disagree | 53 (29.4) | |  | 3 (37.5) | 4 (28.6) | 46 (29.1) |  |
| Disagree | 45 (25.0) | |  | 2 (25.0) | 0 (0.0) | 43 (27.2) |  |
| Neither agree nor disagree (neutral) | 29 (16.1) | |  | 1 (12.5) | 2 (14.3) | 26 (16.5) |  |
| Agree | 42 (23.3) | |  | 2 (25.0) | 6 (42.9) | 34 (21.5) |  |
| Strongly agree | 11 (6.1) | |  | 0 (0.0) | 2 (14.3) | 9 (5.7) |  |
| Missing | 48 | |  | 11 | 28 | 9 |  |
| **I would recommend 12 sessions of weekly percutaneous tibial nerve stimulation (PTNS).** | | | | | | |  |
| Strongly disagree | 32 (17.8) | |  | 1 (12.5) | 3 (21.4) | 28 (17.7) | 0.8260 |
| Disagree | 50 (27.8) | |  | 4 (50.0) | 4 (28.6) | 42 (26.6) |  |
| Neither agree nor disagree (neutral) | 45 (25.0) | |  | 2 (25.0) | 4 (28.6) | 39 (24.7) |  |
| Agree | 35 (19.4) | |  | 1 (12.5) | 1 (7.1) | 33 (20.9) |  |
| Strongly agree | 18 (10.0) | |  | 0 (0.0) | 2 (14.3) | 16 (10.1) |  |
| Missing | 48 | |  | 11 | 28 | 9 |  |
| **I would recommend talking with the physician prescribing the Donepezil/Aricept.**  .. | | | | | | |  |
| Strongly disagree | 38 (21.1) | |  | 1 (12.5) | 2 (14.3) | 35 (22.2) | 0.3931 |
| Disagree | 44 (24.4) | |  | 3 (37.5) | 3 (21.4) | 38 (24.1) |  |
| Neither agree nor disagree (neutral) | 40 (22.2) | |  | 0 (0.0) | 5 (35.7) | 35 (22.2) |  |
| Agree | 45 (25.0) | |  | 4 (50.0) | 2 (14.3) | 39 (24.7) |  |
| Strongly agree | 13 (7.2) | |  | 0 (0.0) | 2 (14.3) | 11 (7.0) |  |
| Missing | 48 | |  | 11 | 28 | 9 |  |
| **I would discuss different types of continence care products with the patient and her husband.** | | | | | | |  |
| Strongly disagree | 3 (1.7) | |  | 0 (0.0) | 1 (7.1) | 2 (1.3) | 0.3482 |
| Disagree | 12 (6.7) | |  | 0 (0.0) | 0 (0.0) | 12 (7.6) |  |
| Neither agree nor disagree (neutral) | 29 (16.1) | |  | 1 (12.5) | 3 (21.4) | 25 (15.8) |  |
| Agree | 75 (41.7) | |  | 6 (75.0) | 4 (28.6) | 65 (41.1) |  |
| Strongly agree | 61 (33.9) | |  | 1 (12.5) | 6 (42.9) | 54 (34.2) |  |
| Missing | 48 | |  | 11 | 28 | 9 |  |
| **I would tell the husband that I do not think that any treatment is warranted.** | | | | | | |  |
| Strongly disagree | 62 (34.4) | |  | 3 (37.5) | 5 (35.7) | 54 (34.2) | 0.4346 |
| Disagree | 84 (46.7) | |  | 4 (50.0) | 7 (50.0) | 73 (46.2) |  |
| Neither agree nor disagree (neutral) | 22 (12.2) | |  | 0 (0.0) | 1 (7.1) | 21 (13.3) |  |
| Agree | 10 (5.6) | |  | 1 (12.5) | 0 (0.0) | 9 (5.7) |  |
| Strongly agree | 2 (1.1) | |  | 0 (0.0) | 1 (7.1) | 1 (0.6) |  |
| Missing | 48 | |  | 11 | 28 | 9 |  |
| **I would make a referral to a social worker.** | | | | | | | |
| Strongly disagree | | 13 (7.2) |  | 0 (0.0) | 1 (7.1) | 12 (7.6) | 0.4726 |
| Disagree | | 24 (13.3) |  | 2 (25.0) | 1 (7.1) | 21 (13.3) |  |
| Neither agree nor disagree (neutral) | | 37 (20.6) |  | 1 (12.5) | 5 (35.7) | 31 (19.6) |  |
| Agree | | 66 (36.7) |  | 4 (50.0) | 2 (14.3) | 60 (38.0) |  |
| Strongly agree | | 40 (22.2) |  | 1 (12.5) | 5 (35.7) | 34 (21.5) |  |
| Missing | | 48 |  | 11 | 28 | 9 |  |
| **I would connect the husband with support groups.** | | | | | | | 0.4500 |
| Strongly disagree | | 8 (4.4) |  | 0 (0.0) | 0 (0.0) | 8 (5.1) |  |
| Disagree | | 26 (14.4) |  | 2 (25.0) | 0 (0.0) | 24 (15.19) |  |
| Neither agree nor disagree (neutral) | | 50 (27.8) |  | 3 (37.5) | 5 (35.7) | 42 (26.58) |  |
| Agree | | 59 (32.8) |  | 3 (37.5) | 4 (28.6) | 52 (32.91) |  |
| Strongly agree | | 37 (20.6) |  | 0 (0.0) | 5 (35.7) | 32 (20.25) |  |
| Missing | | 48 |  | 11 | 28 | 9 |  |
| **I would connect the patient with support groups.** | | | | | | | 0.7910 |
| Strongly disagree | | 26 (14.4) |  | 2 (25.0) | 3 (21.4) | 21 (13.3) |  |
| Disagree | | 57 (31.7) |  | 3 (37.5) | 3 (21.4) | 51 (32.3) |  |
| Neither agree nor disagree (neutral) | | 55 (30.6) |  | 3 (37.5) | 4 (28.6) | 48 (30.4) |  |
| Agree | | 28 (15.6) |  | 0 (0.0) | 2 (14.3) | 26 (16.5) |  |
| Strongly agree | | 14 (7.8) |  | 0 (0.0) | 2 (14.3) | 12 (7.6) |  |
| Missing | | 48 |  | 11 | 28 | 9 |  |
| **I would ask other members of the healthcare team to follow.** | | | | | | | 0.1984 |
| Strongly disagree | | 18 (10.0) |  | 0 (0.0) | 1 (7.1) | 17 (10.8) |  |
| Disagree | | 41 (22.8) |  | 1 (12.5) | 0 (0.0) | 40 (25.3) |  |
| Neither agree nor disagree (neutral) | | 37 (20.6) |  | 3 (37.5) | 4 (28.6) | 30 (19.0) |  |
| Agree | | 60 (33.3) |  | 4 (50.0) | 5 (35.7) | 51 (32.3) |  |
| Strongly agree | | 24 (13.3) |  | 0 (0.0) | 4 (28.6) | 20 (12.7) |  |
| Missing | | 48 |  | 11 | 28 | 9 |  |
| **It is my responsibility as a doctor to treat the patient, not the caregiver [husband].** | | | | | | | 0.4634 |
| Strongly disagree | | 22 (13.0) |  | 2 (33.3) | 2 (20.0) | 18 (11.8) |  |
| Disagree | | 64 (37.9) |  | 3 (50.0) | 3 (30.0) | 58 (37.9) |  |
| Neither agree nor disagree (neutral) | | 36 (21.3) |  | 0 (0.0) | 2 (20.0) | 34 (22.2) |  |
| Agree | | 35 (20.7) |  | 1 (16.7) | 1 (10.0) | 33 (21.6) |  |
| Strongly agree | | 12 (7.1) |  | 0 (0.0) | 2 (20.0) | 10 (6.5) |  |
| Missing | | 59 |  | 13 | 32 | 14 |  |
| **It is my responsibility as a doctor to treat the patient and to alleviate the caregiver’s burden.** | | | | | | | 0.9645 |
| Strongly disagree | | 2 (1.2) |  | 0 (0.0) | 0 (0.0) | 2 (1.32) |  |
| Disagree | | 1 (0.6) |  | 0 (0.0) | 0 (0.0) | 1 (0.66) |  |
| Neither agree nor disagree (neutral) | | 13 (7.7) |  | 0 (0.0) | 0 (0.0) | 13 (8.55) |  |
| Agree | | 96 (57.1) |  | 3 (50.0) | 6 (60.0) | 87 (57.24) |  |
| Strongly agree | | 56 (33.3) |  | 3 (50.0) | 4 (40.0) | 49 (32.24) |  |
| Missing | | 60 |  | 13 | 32 | 15 |  |
| **The fact that the caregiver [husband] is overwhelmed factored into my treatment decision/s.** | | | | | | | 0.0236 |
| Strongly disagree | | 1 (0.6) |  | 0 (0.0) | 0 (0.0) | 1 (0.7) |  |
| Disagree | | 1 (0.6) |  | 0 (0.0) | 1 (10.0) | 0 (0.0) |  |
| Neither agree nor disagree (neutral) | | 10 (6.0) |  | 0 (0.0) | 0 (0.0) | 10 (6.6) |  |
| Agree | | 102 (60.7) |  | 3 (50.0) | 6 (60.0) | 93 (61.2) |  |
| Strongly agree | | 54 (32.1) |  | 3 (50.0) | 3 (30.0) | 48 (31.6) |  |
| Missing | | 60 |  | 13 | 32 | 15 |  |
| **The fact that the husband considered placing the patient in a nursing facility due to her incontinence factored into my treatment decision/s.** | | | | | | | 0.0134 |
| Strongly disagree | | 3 (1.8) |  | 0 (0.0) | 1 (10.0) | 2 (1.3) |  |
| Disagree | | 11 (6.5) |  | 0 (0.0) | 3 (30.0) | 8 (5.2) |  |
| Neither agree nor disagree (neutral) | | 21 (12.4) |  | 1 (16.7) | 1 (10.0) | 19 (12.4) |  |
| Agree | | 74 (43.8) |  | 5 (83.3) | 2 (20.0) | 67 (43.8) |  |
| Strongly agree | | 60 (35.5) |  | 0 (0.0) | 3 (30.0) | 57 (37.3) |  |
| Missing | | 59 |  | 13 | 32 | 14 |  |
| **I felt adequately prepared by training to care for patients with dementia/cognitive impairment and bladder-related issues.** | | | | | | | 0.1127 |
| Yes | | 73 (44.5) |  | 2 (40.0) | 2 (25.00) | 69 (45.7) |  |
| Somewhat | | 74 (45.1) |  | 3 (60.0) | 3 (37.50) | 68 (45.0) |  |
| No | | 17 (10.4) |  | 0 (0.0) | 3 (37.50) | 14 (9.3) |  |
| Missing | | 64 |  | 14 | 35 | 15 |  |
| **I felt adequately prepared by training to care for complex situations related to caregivers of patients with dementia/cognitive impairment and bladder-related issues.** | | | | | | | 0.6303 |
| Yes | | 50 (30.3) |  | 2 (40.0) | 1 (12.5) | 47 (30.9) |  |
| Somewhat | | 81 (49.1) |  | 3 (60.0) | 5 (62.5) | 73 (48.0) |  |
| No | | 34 (20.6) |  | 0 (0.0) | 2 (25.0) | 32 (21.0) |  |
| Missing | | 63 |  | 14 | 34 | 15 |  |
